# Loss of Niemann Pick type C proteins 1 and 2 greatly enhances HIV infectivity and is associated with accumulation of HIV Gag and cholesterol in late endosomes/lysosomes

**DOI:** 10.1186/1743-422X-9-31

**Published:** 2012-01-24

**Authors:** Ebony M Coleman, Tiffany N Walker, James EK Hildreth

**Affiliations:** 1 Center for AIDS Health Disparities Research, Meharry Medical College, Nashville, TN, USA; 2 Department of Molecular and Cellular Biology, University of California, Davis, CA, USA

## Abstract

**Background:**

Cholesterol pathways play an important role at multiple stages during the HIV-1 infection cycle. Here, we investigated the role of cholesterol trafficking in HIV-1 replication utilizing Niemann-Pick Type C disease (NPCD) cells as a model system.

**Results:**

We used a unique NPC2-deficient cell line (NPCD55) that exhibited Gag accumulation as well as decreased NPC1 expression after HIV infection. Virus release efficiency from NPCD55 cells was similar to that from control cells. However, we observed a 3 to 4-fold enhancement in the infectivity of virus released from these cells. Fluorescence microscopy revealed accumulation and co-localization of Gag proteins with cholesterol in late endosomal/lysosomal (LE/L) compartments of these cells. Virion-associated cholesterol was 4-fold higher in virions produced in NPCD55 cells relative to virus produced in control cells. Treatment of infected NPCD55 cells with the cholesterol efflux-inducing drug TO-9013171 reduced virus infectivity to control levels.

**Conclusions:**

These results suggest cholesterol trafficking and localization can profoundly affect HIV-1 infectivity by modulating the cholesterol content of the virions.

## Background

Cellular cholesterol plays a critical role in various stages of the HIV-1 replication cycle. HIV-1 fusion, entry, assembly, and budding occur at cholesterol-enriched microdomains called lipid rafts [1-4]. The HIV-1 accessory protein, Nef, has been shown to induce many genes involved in cholesterol biosynthesis and homeostasis [5,6]. Depletion of virion-associated cholesterol by beta-cyclodextrin compromises viral structural integrity and significantly decreases both the quantity and infectivity of virions released from infected cells [7,8]. Treatment of HIV particles with cholesterol-sequestering compounds inhibits virus entry into host cells [9,10].

Previous studies have shown that Nef inhibits the activity of ATP-binding cassette transporter A1 (ABCA1) in HIV-infected macrophages. The inhibition of ABCA1 leads to suppression of cholesterol efflux and an accumulation of intracellular cholesterol [11]. In turn, this effect increases the cholesterol content of the virions. The proteins implicated in Niemann-Pick Type C (NPC) disease, NPC1 and NPC2, are responsible for the egress of intracellular cholesterol and glycosphingolipids from late endosomal/lysosomal (LE/L) compartments [12-14]. Patients carrying mutations in either NPC1 or NPC2 display phenotypes that are clinically and biochemically indistinguishable. The two NPC proteins have been recently shown to function in the same pathway [15-17]. The hallmark phenotype of cells deficient in either NPC1 or NPC2 is accumulation of unesterified LDL-derived cholesterol in LE/L compartments [18-21].

HIV-1 Gag accumulates in the cholesterol-laden LE/L compartments of NPC1-deficient cells and virus release is dramatically reduced [22]. LE compartments can serve as sites for HIV-1 assembly and budding [23-26] and host proteins that reside in these compartments are incorporated into newly released virions [27,28]. Given that NPC proteins mediate cholesterol transport from the LE/L compartment to other compartments, we sought to utilize NPC disease as a model for investigating whether this cholesterol transport pathway is essential for HIV-1 assembly and release. Fibroblasts from four donors of each cell type- normal, NPC1-deficient (NPC1D), and NPC2-deficient (NPC2D), were used to study HIV-1 replication. Cells from one donor (NPCD55) whose HIV replication phenotype was strikingly different from cells of other donors provided a useful tool for our studies. Our findings demonstrate a link between intracellular cholesterol transport and localization and HIV-1 infectivity.

## Results

### Expression levels of HIV-1 Gag and NPC proteins in fibroblasts

**Figure 1:**
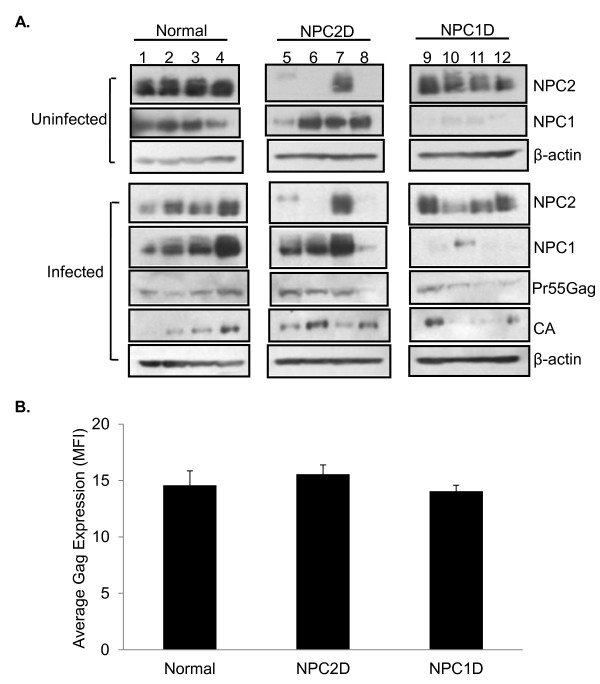
**Protein expression analysis of normal and NPC-deficient cells after HIV-1 infection**. Cells were uninfected or infected with VSVG-HIV-1 and harvested 96 h post-infection. (**A**) NPC2, NPC1, and β-actin protein expression was detected via Western blotting in uninfected and infected cells. Gag expression was also detected in the infected cells. (**B**) At 96 h post infection, cells were fixed and stained with KC57-FITC (anti-Gag). Flow cytometry was performed and mean fluorescence intensity (MFI) is shown.

Because of the inherent cholesterol transport defect in NPCD cells, they were used to examine the impact of reduced cholesterol transport capability on HIV-1 replication. Normal, NPC2D, and NPC1D fibroblasts were infected with the single-cycle HIV-1 VSVG-NL4.3. The VSVG-NL4.3 virus was made by pseudotyping env-deleted NL4.3 with VSV G protein. Gag p55 and p24 expression was measured by Western blot analysis (Figure 1A). Intracellular Gag was measured via flow cytometry and the mean fluorescence intensity (MFI) data showed that across infected cell types there was no significant difference in Gag expression (Figure 1B).

Because of the genetic mutations in NPC2D and NPC1D, we expected NPC2D (Figure 1A, lanes 5-8) and NPC1D (Figure 1A, lanes 9-12) fibroblasts to express much lower levels of NPC2 and NPC1, respectively, when compared to controls (Figure 1A, lanes 1-4). The NPC2 bands observed in lanes 5 and 7 represent mutated forms of protein that are non-functional (Coriell Repository, Camden, NJ). Interestingly, the results in lane 8 show a striking decrease in NPC1 expression upon infection of one of the NPC2D cell lines with HIV-1 (Figure 1A). This result is in contrast to other NPC2D and normal cells that normally show no change or an increase in NPC1 expression upon HIV infection. Normal and NPC2D cells showed approximately a 1:1 ratio of p55 to p24 (Figure 1A, lanes 2-7). Along with cells from normal donors, we included cells from NPC1D donors as controls. In Figure 1A, the results in lane 12 are consistent with our previous findings showing Gag accumulation in cells from this NPC1 donor. The reduction in NPC1 expression upon infection of NPC2D cells in lane 8 of Figure 1A provided a model system to study HIV-1 assembly and release in the context of low or absent expression of both NPC1 and NPC2. In these cells the export of cholesterol from LE/L compartments is presumably very low or completely impaired. Therefore, our studies focused on characterizing the HIV phenotype in this cell line, henceforth designated as NPCD55.

### HIV-1 Gag protein accumulates in cholesterol-laden LE/L compartments of NPCD55 cells

**Figure 2:**
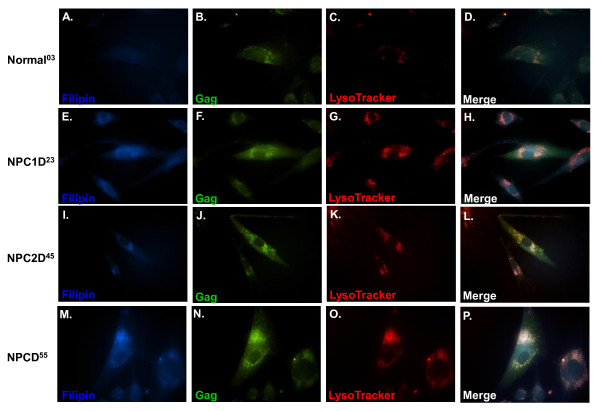
**Gag localizes to LE/L compartments in NPC-deficient cells**. At 96 h post-infection with HIV-1, fibroblasts were stained and analyzed by fluorescence microscopy to assess Gag localization. Intracellular cholesterol (blue) was visualized by staining the cells with filipin dye (**A, E, I, M**). LysoTracker was used to stain late endosomes/lysosomes (red) (**C, G, K, O**) and HIV-1 Gag protein (green) (**B, F, J, N**) was stained with KC57-FITC.

We used dual-label immunofluorescence and filipin staining to visualize Gag, LE/L, and cholesterol in HIV-1-infected cells. Results in infected normal cells showed marginal and diffuse staining of HIV-1 Gag and cholesterol (filipin staining) (Figure 2A-B). Gag showed a high degree of colocalization with cholesterol in LE/L compartments of NPC1D cells (Figure 2H) as previously reported [22]. HIV-1-infected NPC2D cells displayed a Gag localization pattern similar to NPC1D cells (Figure 2L). Gag accumulated in the perinuclear region of the cell and colocalized with cholesterol in LE/L compartments (Figure 2I-L). As in NPC1D and NPC2D cells, Gag colocalized with cholesterol in NPCD55 cells. However, the staining intensity of these two components was much higher in NPCD55 cells (Figure 2M-P). Previous studies have shown that the trafficking of several host proteins is impaired in NPCD cells [29-32]. Our data appear to indicate that like these host proteins, HIV-1 Gag transport is retarded in NPCD55 cells and the protein accumulates in LE/L compartments.

### Cholesterol content of virions released from NPCD55 cells is enhanced

**Figure 3:**
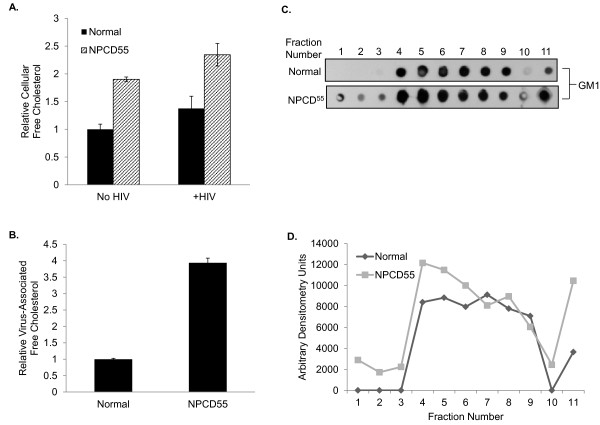
**Free cholesterol and lipid raft content of normal and NPCD55 cells**. (**A**) Cellular cholesterol was measured in uninfected and HIV-1-infected Normal (filled) and HIV-1-infected NPCD55 (slashed) cells by AmplexRed assay. Cholesterol concentration was normalized to total protein. Error bars represent triplicate experiments. Values are relative to the normal uninfected control which was arbitrarily set to 1. (**B**) Virus produced from each cell type was concentrated by centrifugation through a 20% sucrose cushion. The cholesterol content of the virus was measured by AmplexRed assay and input was normalized by p24 concentration. (**C**) Lipid raft fractions were collected for normal and NPCD55 cells and loaded for dot blot analysis. Membranes were probed with cholera toxin B to detect GM1. The samples were normalized by protein concentration and volume. (**D**) Dot intensities for the blot shown were quantified using ChemiDoc XRS and Quantity One software.

Colocalization of Gag with cholesterol in LE/L compartments of NPCD55 cells could promote the incorporation of cholesterol into budding virions. Therefore, we quantified the cholesterol content of virions released from HIV-1-infected NPCD55 and HIV-1-infected normal cells. HIV-1 infection has been shown to increase cholesterol biosynthesis in infected cells [29]. We measured intracellular cholesterol in normal and NPCD55 cells 96 h post-infection. As expected, the intracellular cholesterol content of uninfected NPCD55 cells was much higher than that of normal cells (Figure 3A). Both cell types showed a slight increase in free cholesterol after infection (Figure 3A). Virions released from NPCD55 cells contained approximately 4-fold more cholesterol than virions released from normal cells (Figure 3B). In a recent report describing AnxA6 dependence on Ca^2+ ^for recruitment into lipid rafts in a fibroblast cell line (L1) derived from an NPCD patient, Domon et al. demonstrated an increased GM1 content in the L1 cells [30]. This ganglioside is highly enriched in lipid rafts and serves as a useful marker for lipid rafts in solubilized membrane flotation assays [31,32]. HIV-1 budding and assembly occurs primarily at lipid rafts and Gag has been shown to associate with these sites [33-35]. HIV-1 Nef has been shown to increase HIV-1 infectivity via a lipid raft-dependent mechanism [36,37]. We hypothesized that the enhanced cholesterol content of virions released from NPCD55 cells might be correlated with increased lipid rafts in these cells. In membrane flotation assays, GM1 could be detected as early as fraction 1 of NPCD55 cells whereas GM1 expression was not seen until fraction 4 of normal cells (Figure 3C). A plot of the GM1 staining intensities in the fractions from the flotation assays showed that the overall GM1 content of NPCD55 cells was much greater than that of normal cells (Figure 3D). This result is consistent with a higher content of lipid rafts in NPCD55 cells compared to the control cells.

### Enhanced infectivity of virions produced in NPCD55 cells

**Figure 4:**
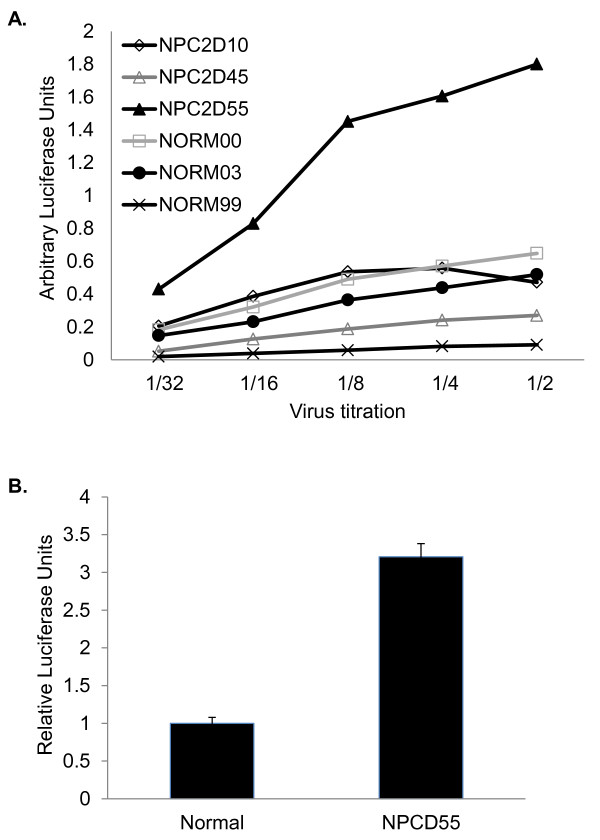
**Infectivity of virus produced from normal and NPCD55 cells**. (**A**) Virus containing supernatants from three normal and NPC2D fibroblasts were titrated onto TZM-bl indicator cells. At 48 h post-infection, the cells were harvested and luciferase assay was performed to measure virus infectivity. Luciferase activity was normalized to virus input. (**B**) TZM-bl cells were infected with 5 ng of purified virus released from Normal and NPCD55 cells. Infectivity was measured by luciferase activity and normalized to Normal cells, which was arbitrarily set to 1. Error bars are representative of three independent infections.

Virion-associated cholesterol is critical for HIV-1 infectivity [38,39]. In light of the results showing increased cholesterol in HIV-1 from NPCD55 cells, we examined the infectivity of virus produced from these cells. HIV-1 was produced in a panel of normal and NPC2D cell lines. The virus-containing supernatants were collected and titrated onto TZM-bl reporter cells. These cells express beta-galactosidase and luciferase under the control of the HIV-1 LTR and can be used to study HIV-1 infectivity. The virus produced in NPCD55 cells showed a striking increase in infectivity compared to virus produced in the other five cell lines (Figure 4A). The much higher infectivity of NPCD55-derived virus was highly reproducible. When TZM-bl cells were infected with purified virus produced in normal and NPCD55 cells, virions from NPCD55 cells yielded 3-fold higher luciferase activity than virions from normal cells (Figure 4B). These results indicate that HIV produced in NPCD55 cells is more highly infectious than virus produced in normal and other NPC2D cells.

### TO-901317 treatment stimulates cholesterol efflux and reduces infectivity of HIV-1 virions produced in NPCD55 cells

**Figure 5:**
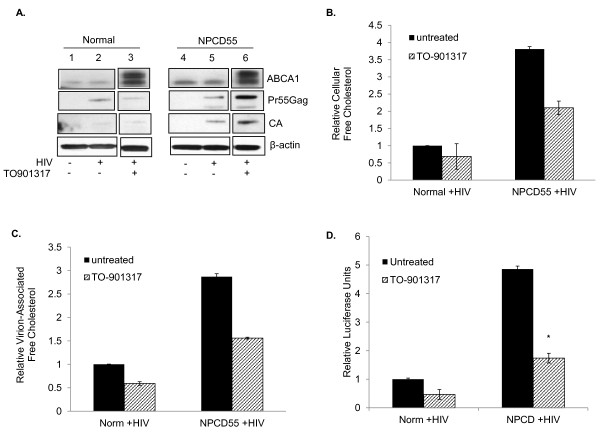
**Induction of cholesterol efflux attenuates the enhancement in HIV-1infectivity in NPCD55 cells**. (**A**) Normal and NPCD55 cells were treated with 5 μM TO-901317 at 24 h post-infection and cultured for 72 h in the presence of the compound. At 96 h post-infection the cells were harvested and ABCA1, Gag, and β-actin expression was detected by Western blotting analysis. All samples were loaded on the same gel. (**B**) AmplexRed assay was performed to measure free cholesterol content of untreated (filled) and TO-901317-treated (slashed) cells. Cholesterol content was normalized to protein concentration. (**C**) AmplexRed assay was performed to measure virion-associated cholesterol from purified virus produced in untreated (filled) and TO-901317-treated (slashed) infected cells. Virion-associated cholesterol content was normalized to p24 concentration. (**D**) TZM-bl reporter assay was performed to measure virus infectivity when cholesterol efflux was induced via TO-901317 stimulation. The ρ values were calculated by performing student T-test (*denotes < 0.05).

Results obtained thus far demonstrate that in HIV-1-infected NPCD55 cells Gag colocalizes with cholesterol in LE/L compartments, virions produced in these cells incorporate more cholesterol, and the virions released are more infectious (Figures 2, 3B and 4B). Earlier studies from our laboratory and others have shown that the cholesterol content of HIV-1 can affect infectivity of the virus [40-42]. Therefore we sought to determine whether enhanced infectivity of HIV-1 from NPCD55 cells was due to higher cholesterol content. To this end, we stimulated cholesterol efflux in normal and NPCD55 cells by treating the cells with the synthetic nonsteroidal LXR agonist, TO-901317. TO-901317 treatment of NPC1-deficient fibroblasts results in an increase in ABCA1 expression, marked increase in cholesterol efflux, and reduction of unesterified cholesterol in the LE/L compartment [43]. We infected normal and NPCD55 cells according to the protocol described above except that at 24 h post-infection cells were cultured in complete medium or medium supplemented with 5 μM TO-901317 for 72 hrs. ABCA1 expression was significantly enhanced upon TO-901317 treatment of both normal and NPCD55 cells (Figure 5A, lanes 3 and 6). Induction of cholesterol efflux resulted in increased presence of both unprocessed (p55) and processed (p24) gag in HIV-1-infected NPCD55 cells, but not in HIV-1-infected normal cells (Figure 5A, lanes 5-6). Stimulation of cholesterol efflux resulted in a 20% reduction of free cholesterol in HIV-1-infected normal cells and almost 2-fold decrease in HIV-1-infected NPCD55 cells (Figure 5B). Similar levels of decrease were found when cholesterol was measured in HIV-1 released from normal and NPCD55 cells (Figure 5C). We next examined the infectivity of virus released from control and TO-901317-treated cells. As seen in Figure 5D, TO-901317 treatment of NPCD55 cells reduced the infectivity of released HIV-1 by more than 2-fold, down to a level close to that of virus released from control cells. Based on these data we conclude that the enhanced infectivity of virus released from NPCD55 cells is due largely to the increased amount of free cholesterol in the LE/L compartments in these cells that results in higher virion-associated cholesterol. Our findings are in agreement with previous work in which the induction of ABCA1 by TO-901317 resulted in inhibition of HIV-1 replication [44].

## Discussion

We have identified an NPC2 deficient cell line (NPCD55) that loses expression of NPC1 upon infection by HIV-1. This allowed us to examine the role of a critical cholesterol homeostatic pathway, export of cholesterol from endosomes, in HIV-1 replication. Our data show that when both major proteins involved in this pathway are poorly expressed, HIV-1 acquires much more cholesterol and is much more infectious. The fluorescence microscopy analysis demonstrates that HIV-1 Gag proteins localize to cholesterol-laden LE/L compartments in NPCD55 cells. The strong co-localization of Gag with cholesterol to LE/L compartments of NPCD55 cells correlated with a much higher cholesterol content in virions released from these cells. These data are consistent with prior studies showing that HIV assembly occurs on lipid raft membranes as are found in late endosomes [3,25,45]. Induction of cholesterol efflux showed that free cholesterol levels within the LE/L compartments in NPCD55 cells was likely responsible for the increased cholesterol content in virions produced in these cells. NPCD55 cells not only had increased free cholesterol, but also showed evidence of a marked enhancement of lipid rafts. Prior studies provide evidence that lipid rafts accumulate in LE/L compartments in NPCD cells [46,47]. Neither an overall increase in intracellular cholesterol nor increased lipid raft content alone would account for such a significant increase in virus infectivity as observed in virus produced from NPCD55 cells. However, the correct subcellular localization of unesterified cholesterol is probably critical to HIV infectivity. Since the cell's LE compartments are major sites of HIV-1 assembly and budding, the accumulation of cholesterol and lipid rafts in LE/L compartments may explain the enhanced infectivity of HIV-1 released from NPC2D55 cells.

Our work initially included cells from four NPC2D donors. Because NPC1 and NPC2 function in the same pathway and HIV-1 replication is inhibited when NPC1 is absent, we wanted to test whether HIV-1 replication was also inhibited in the absence of NPC2. Interestingly, we did not observe the drastic reduction in virus release from NPC2D cells as we had previously demonstrated with NPC1D cells [22]. When NPC2D cells were infected, virus release efficiency was similar to controls (data not shown). The infectivity of virions produced in NPC2D cells was slightly higher than the infectivity of virions produced in control cells; however, the infectivity of virions produced in NPCD55 cells was strikingly higher than that of virus from control cells and from other NPC2D cell lines. We then focused our studies on utilizing NPCD55 cells to further define how cholesterol transport pathways contribute to HIV-1 infectivity. Since all NPC2D cells lacked a functional NPC2 protein and displayed the characteristic NPC phenotype, we expected that the infectivity of virions produced from these cells would be similar.

The NPC1 gene contains sterol response elements and expression of the protein is modulated by SREBPs [48-50]. Thus, the decrease in NPC1 expression in NPCD55 cells after infection with HIV-1 was unexpected since sterol response genes are upregulated after HIV-1 infection [5,29]. Interestingly, NPC1 expression was increased as expected in other cell lines after HIV infection. It is not clear why NPC1 expression was lost after HIV-1 infection of NPCD55 cells. Mutations in the promoter region may have affected the ability of SREBPs to modulate NPC1 expression. Previous reports demonstrate that the intronic microRNA, miR-33, is located within the gene encoding SREBP-2 and that these molecules are coordinately regulated [51,52]. Further, miR-33 inhibits ABCA1 mediated cholesterol efflux and strongly suppresses NPC1 protein expression. Therefore, the decreased expression of NPC1 could be miR33-mediated via induction of SREBP expression upon HIV-1 infection of NPCD55 cells. HIV-1 Nef has been shown to impair cholesterol efflux by directly interacting with ABCA1 and dowregulating ABCA1 expression [11]. Though the mechanism is unclear, the interaction between Nef and ABCA1 is not required for post-translational degradation of ABCA1 [53]. NPC1 protein expression could also be decreased upon HIV-1 infection in a manner similar to ABCA1 downregulation. However, this does not explain why loss of NPC1 did not occur in other HIV-1-infected NPC2D cells.

Another possibility for differences among NPC2D cells is the level of cholesterol esterification activity among the cells. NPCD55 cells were characterized as having undetectable cholesterol esterification (Coriell Institute for Medical Research, Coriell Cell Repositories, unpublished), whereas cells from other NPC2D donors show some level of cholesterol esterification activity. The ratio of free cholesterol to cholesterol esters may be different between NPCD55 cells and other NPC2D cells, with NPCD55 cells containing more unesterified cholesterol. This is important since unesterified cholesterol is incorporated into budding virions, and the lack of esterase activity would enhance the pool of unesterified cholesterol. Free cholesterol, but not cholesterol esters, can also suppress induction of sterol response genes. The infectivity of HIV produced in other NPC2D cell lines was generally similar to that of virus produced in normal cell lines. Thus the cholesterol accumulation phenotype alone of NPCD does not appear to cause changes in HIV-1 infectivity. The dramatic enhancement of infectivity in virus produced in NPCD55 cells was associated with a remarkable and unique co-localization of free cholesterol and HIV-1 Gag protein. This probably produced an ideal setting for incorporation of the critical lipid by the virus. However, we cannot rule out the possibility that NPCD55 cells may have other unidentified genetic mutations that could affect the function of other proteins involved in regulating HIV-1 infectivity.

In summary, our findings demonstrate that defects in the NPC1/NPC2 pathway of cholesterol efflux from late endosomes can profoundly affect HIV-1 infectivity and highlight the contribution of intracellular cholesterol transport and localization to virus infectivity.

## Methods

### Cells

Fibroblasts from healthy- normal donors (GM00500, GM09503, GM00409, and GM00499) as well as patients carrying mutations in the NPC1 (GM00110, GM17913, GM17921, GM03123) or NPC2 (GM17910, GM18439, GM18445, GM18455) genes were obtained from Coriell Repositories (Coriell Institute for Medical Research, Camden, NJ). The fibroblasts were maintained in Dulbecco modified Eagle medium (Gibco-BRL/Life Technologies, Gaithersburg, MD) supplemented with 15% defined fetal bovine serum (FBS-D) (HyClone, Logan, UT), L-glutamine, and 10 mM HEPES (pH 7.2). Cell viability was assessed by trypan blue exclusion. TZM-bl HIV-1 indicator cells were obtained from the NIH AIDS Research and Reference Reagent Program (Germantown, MD) and maintained in DMEM supplemented with 10% fetal calf serum (FCS), L-glutamine, and 10 mM HEPES (pH 7.2), and 100 U/ml penicillin and streptomycin (cDMEM). 293 T human embryonic kidney cells were maintained in DMEM supplemented with L-glutamine, and 10 mM HEPES (pH 7.2), and 10% FCS (HyClone, Logan, UT).

### Virus

Vesicular stomatitis virus envelope glycoprotein G (VSV-G)-pseudotyped HIV-1 was prepared using 293T cells. Cells were co-transfected with pNL4.3-GFP plasmid (kind gift from Dr. Robert Silicano, Johns Hopkins School of Medicine) and the VSV-G expression vector pHEF-VSV-G using the calcium phosphate transfection method. Briefly, at 48 h post-transfection the culture supernatants containing virus particles were collected and filtered through a 0.45 μm filter. The virus was quantified by p24 ELISA and used for direct infection of fibroblasts.

### Western blot analysis

Intracellular protein expression was analyzed by standard Western blot using the NuPAGE gel electrophoresis system (Invitrogen, Carlsbad, CA). Briefly, cells were lysed on ice for 30 min in RIPA buffer (50 mM Tris-HCl, Adjust to pH 7.41, 50 mM NaCl, 1 mM PMSF, 1 mM EDTA, 5 μg/ml protinin, 5 μg/ml Leupeptin, 1% Triton x-100, 1% Sodium deoxycholate, 0.1% SDS) supplemented with protease inhibitors (Roche catalogue no. 1873580). Lysates were then clarified of cell debris by centrifugation at 13,000 rpm at 4°C for 20 min. Lysates were run on 4-12% Bis-Tris gels, and then transferred onto nitrocellulose using a semi-dry transfer apparatus (BioRad). Membranes were blocked for 30 min in Superblock (Invitrogen, Carlsbad, CA) before probing with primary antibodies. Following washing 3 times in phosphate buffered saline containing 0.05% Tween-20 (PBS-T), the membranes were probed with HRP conjugated secondary antibodies. Chemiluminescent substrate (ECL, GE Healthcare Life Sciences) was used for detection.

### Antibodies

The antibodies used in this study were rabbit polyclonal antibodies: anti-NPC1 (Novus Biologicals), anti-ABCA1 (Novus Biologicals), and anti-β-actin (Sigma-Aldrich). Rabbit polyclonal antibody against NPC2 was a kind gift from Dr. Peter Lobel (UMDNJ-Robert Wood Johnson Medical School, Piscataway, NJ). Gag monoclonal anti-p24 was purchased from Millipore (Temecula, CA). Secondary antibodies (HRP conjugated goat anti-rabbit or mouse heavy- and light-chain specific) were purchased from Jackson ImmunoResearch Laboratories (Westgrove, PA).

### Infection of fibroblasts

Normal and NPC-deficient fibroblasts were seeded (3 × 10^5 ^cells) in 10-cm culture plates in cDMEM. Cells were allowed to grow for 12-16 h and infected with 3 μg of VSV G-NL4.3, normalized for HIV-1 capsid p24. After 24 h, the cells were washed to remove virus and cultured in fresh media. At 96 h post infection, the cells and supernatants were harvested. Supernatants were collected and stored at -80°C for further analysis. Cells were trypsinized, washed 3 times in PBS, and prepared for Western blot or flow cytometry analysis. Infection efficiencies were determined by flow cytometry. Briefly, cells were fixed with 2% paraformaldehyde, permeabilized using 0.1% saponin, and stained with fluorophore-conjugated antibodies. Stained cells were analyzed using the Becton FACSCalibur flow cytometer (Cell Quest software). Virus produced from these cells was quantified by p24 ELISA and normalized to the percentage of cells that were positive for Gag.

### Cholesterol efflux

Fibroblasts were cultured and infected with VSVG-NL4.3 as previously described. At 24 h post infection, the input virus was removed and the cells were washed 3 times in PBS. Cells were then cultured in medium alone, medium supplemented with 15% Lipoprotein Deficient Serum (LPD-S), or medium containing 5 μM TO-901317 (Sigma-Aldrich, St. Louis, MO). Cells and supernatants were harvested 96 h post infection.

### Virus titer determination

Virus released from infected cell cultures was measured using an ELISA developed in our laboratory to measure viral p24 antigen (sensitivity 50-2000 pg/mL).

### Cell staining and immunofluorescence assay

Cells were grown on 35-mm glass bottom dishes (MatTek Corporation, Ashland, MA). For LysoTracker Red staining, cells were incubated for 2 h at 37°C with 75 nM LysoTracker Red DND-99 and then fixed for 15 min in 2% paraformaldehyde in PBS. The cells were permeabilized with 5% normal goat serum in BD Cytofix/Cytoperm solution (BD Biosciences, San Diego, CA). Gag and cholesterol staining was performed using KC57-FITC (Beckman Coulter, Inc., Fullerton, CA) and filipin (Sigma-Aldrich, St. Louis, MO) respectively. The reagents were diluted in BD Cytofix/Cytoperm solution and added to the cells for 30 min at room temperature. Cells were washed 2 times in PBS and anti-fade solution was applied before imaging. Photographs were taken using a Nikon TE2000 wide-field microscope (Nikon Instruments, Melville, NY) with a 40X oil objective. The FITCx was utilized to eliminate cross excitation between DAPI and FITC.

### Flow cytometry

After cells were stained and fixed as previously described, the cells were washed in PBS and resuspended in 1 mL FACS buffer (1XPBS, 5% FCS, and 0.1% sodium azide). The cells were then analyzed on a FACSCalibur (Becton Dickson) flow cytometer.

### Infectivity assay

TZM-bl cells were plated in 96-well microtiter plate at a density of 12.5 × 10^3^/100 μl. After 24 h, cells were infected for 12 h with virus generated from infected fibroblasts. The input virus was removed and the cells were washed 3 times in PBS and maintained under normal culture conditions. At 24 h-post infection, cells were washed 3 times in PBS and luciferase assay was performed using a luciferase reporter gene assay system (Luc-Screen kit; Applied Biosystem, Foster City, CA). Relative luciferase units were normalized to input p24 values.

### Cholesterol quantification

Cellular and virion associated cholesterol was measured using the Amplex^®^Red Cholesterol Assay Kit (Invitrogen, Carlsbad, CA). Samples were processed in accordance with the manufacturer protocol. To measure unesterified cholesterol, the cholesterol esterase enzyme was eliminated from the reaction mixture. Cholesterol content for cells and virions was normalized to protein and p24 concentration. Protein concentrations of cell lysates were determined by BCA assay (Fisher, Waltham, MA). Virus p24 concentrations were measured by p24 ELISA.

### Isolation of lipid rafts

Lipid rafts were isolated from cell lysates as previously described by Popik et al. [54]

## Competing interests

The authors declare that they have no competing interests.

## Authors' contributions

EMC and JEKH designed, coordinated the study, and drafted the manuscript. EMC carried out the experiments. TNW contributed to the production of VSVG-NL4.3, performed flow cytometry analysis, and performed initial experiments involving the infection and processing of fibroblasts from the twelve donors. All authors read and approved the final manuscript.
